# Intestinal Peripheral T-Cell Lymphoma in a Patient with Ankylosing Spondylitis Under Treatment with Infliximab: A Case Report and Review of the Literature

**DOI:** 10.31138/mjr.33.2.247

**Published:** 2022-06-30

**Authors:** Firdevs Ulutaş, Cansu Korkmaz, Halil Yılmaz, Duygu Akça, Erdem Çomut, Mustafa Çelık, Veli Çobankara

**Affiliations:** 1 Division of Rheumatology, Department of Internal Medicine, Pamukkale University Faculty of Medicine, Denizli, Turkey**,**; 2 Department of Internal Medicine, Pamukkale University Faculty of Medicine, Denizli, Turkey**,**; 3 Division of Gastroenterology, Department of Internal Medicine, Pamukkale University Faculty of Medicine, Denizli, Turkey**,**; 4 Department of Pathology, Pamukkale University Faculty of Medicine, Denizli, Turkey

**Keywords:** ankylosing spondylitis, Crohn’s disease, lymphoma, inflammation

## Abstract

**Background:**

Recent literature involves many cases with lymphoma and ankylosing spondylitis (AS) with or without the use of TNF inhibitors. Herein, we report a patient, a 56-year-old Human Leukocyte Antigen-B27 (HLA-B27) positive man with four years history of AS who was still under treatment with infliximab with clinical remission. He was admitted with a new-onset, 6-week history of bloody diarrhoea with mucus, abdominal pain, fever, and weight loss. An ileocolonoscopy showed linear ileocecal valve ulcers. Histopathological findings of ileocecal valve ulcers revealed peripheral T-cell lymphoma of the small intestine. Infliximab was interrupted because of the possible progression of the lymphoma**.**

**Methods:**

We aimed to emphasize the underlying potential pathogenic mechanisms and to review the related literature. A literature search was conducted in the PubMed database between January 1980 and November 2020. The keywords including ‘ankylosing spondylitis’ and ‘lymphoma’ were used.

**Conclusion:**

TNFi use, immunosuppression, and chronic inflammation may be related to the development of lymphoma in chronic inflammatory diseases. Ileocecal valve involvement should not be interpreted as inflammatory bowel disease, infection, or vasculitis in the presence of red flags.

## MAIN POINTS

- Chronic inflammation is one of the major predisposing contributors to lymphoma.
- TNFi use and immunosuppression may be related to the development of lymphoma in inflammatory diseases.
- Ileocecal valve involvement should not be interpreted as inflammatory bowel disease, infection, or vasculitis in the presence of red flags.

## INTRODUCTION

Chronic inflammation and/or continuous antigen stimulation are major predisposing contributors to lymphoma. Activation of humoral immunity plays a key role in pathogenesis.^1^ This inflammatory status may precede as well as follow the tumour development. Several autoinflammatory diseases such as rheumatoid arthritis and Sjogren’s syndrome are well-known to be related to the development risk of lymphoma.^2^ A few points are still uncertain in the pathogenesis. Although additive effects of smoking, Epstein-Barr virus (EBV), and/or human immunodeficiency virus (HIV) infection and immunosuppressive drugs are noticed in the recent literature, there is quite a heterogeneity in the development of lymphoma in some patients.^3^ Tumour necrosis factor-alpha (TNF-α) is the main cytokine that plays a culprit role in chronic inflammatory diseases. It has a principal regulator role of pro-inflammatory status via its effects on inflammatory cells, adhesion molecules, and cytokine production.^4^ Suppression of this cytokine with anti-TNF agents brought a new era in the rheumatology practice. Infliximab is a mouse/human chimeric monoclonal antibody that neutralises the activity of TNF-α, fixes complement, and leads to the death of inflammatory cells expressing TNF-α.^5^ Mariette X et al. noted a two to threefold high risk of lymphoma in patients receiving anti-TNF therapy such as expected in severe inflammatory diseases, and also revealed a higher risk with monoclonal-antibodies than with soluble-receptor therapy among tumour necrosis factor inhibitors (TNFi).^6^ The association between ankylosing spondylitis (AS) and lymphoma is not clear. A comprehensive systematic review and meta-analysis revealed an increased risk of malignancies of the gastrointestinal system and haematological system including multiple myelomas and lymphomas in AS patients in 2016.^7^ Besides, Askling J et al. did not find an increased lymphoma risk among hospitalized AS patients in contrast to rheumatoid arthritis patients in the Swedish population.^8^ The same uncertainty exists concerning TNFi use. A small cohort including low numbers of patients with lymphoma and AS showed no difference of lymphoma incidence between TNFi-exposed versus TNFi-naive AS patients.^9^ Hellgren K et al. announced that there is no increased risk of any malignancy related to TNFi use in AS patients in addition to lymphoma.^10^ However, literature involves many AS patients who developed Hodgkin’s or Non-Hodgkin’s lymphoma after TNFi use.^11^

Herein, we report an intestinal peripheral T-cell lymphoma in a patient with ankylosing spondylitis under treatment with infliximab. We aimed to emphasise the underlying potential pathogenic mechanisms and to review the related literature.

## CASE PRESENTATION

A 56-year-old HLA-B27-positive man was diagnosed with AS in 2016, characterised by chronic inflammatory back pain with the onset of age 30 and early morning stiffness of around 4 hours, and overt bilateral sacroiliitis on X-ray of sacroiliac joints (SIJs). He was on the regular follow-up in the rheumatology outpatient clinic of the tertiary health centre. He was currently under treatment with infliximab (400 mg/6 weeks) in addition to regular physical rehabilitation for four years without any low back pain and morning spinal stiffness. He was admitted with a new-onset, 6-week history of bloody diarrhoea with mucus and abdominal pain. He was also complaining of 14 kg weight loss in the last month, night sweats, and a metallic taste in the mouth. He had no risk factors including familial or personal history of any malignancy or inflammatory bowel disease (IBD), smoking, EBV infection, and additional immunosuppressant use except infliximab.

On his physical examination, he had normal vital signs, abdominal tenderness in the right lower abdominal quadrant. Lymph nodes were not palpable in the neck, axilla, and inguinal regions. His spinal movements had limited anterior and lateral flexion and extension in addition to restricted Schober’s test and chest expansion. Laboratory analysis revealed microcytic normochromic anaemia with a haemoglobin level of 10.7 g/dL, neutrophilic leucocytosis of 15,500/μL, an erythrocyte sedimentation rate (ESR) of 78 mm/h, and a C-reactive protein (CRP) level 5 times the upper limit of normal (ULN). Peripheral blood smears showed no atypic and/or blastic cells. Stool microbiological investigations were negative for ova and parasites, Giardia antigen, and bacterial cultures. Laboratory and histologic examinations ruled out intestinal tuberculosis and cytomegalovirus (CMV) infection.

**Figure 1.**
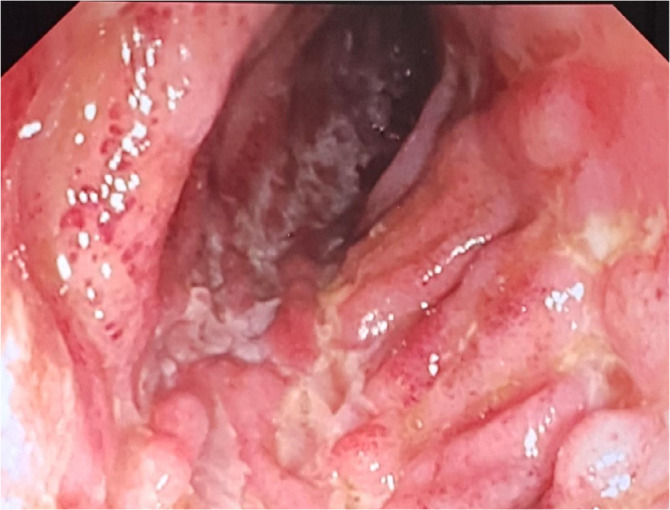
An ileocolonoscopy; linear ileocecal valve ulcers, erythema and losss of vascular pattern of the mucosa because of intestinal wall edema.

**Figure 2.**
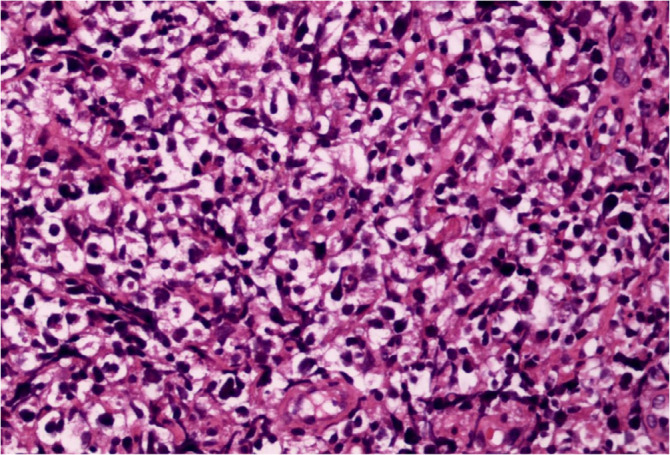
Large lympoid cells wit prominent clear cell features in periperal T-cell lympoma, NOS (H&E, x400).

**Figure 3.**
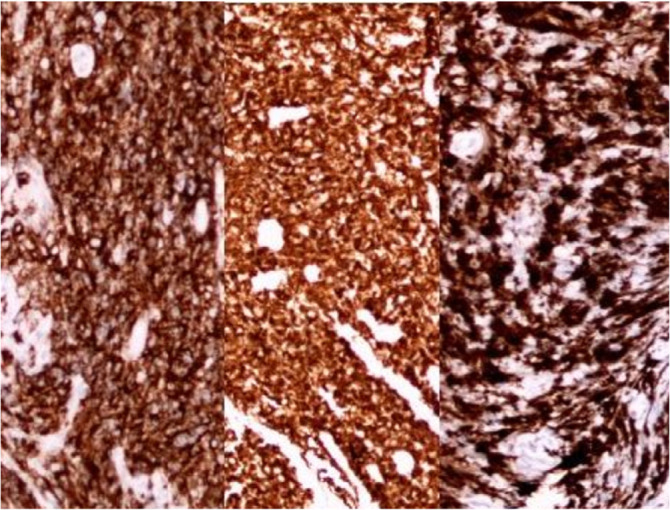
Neoplastic cells express CD4 (a), CD30 (B) and GATA-3 (C) (x200).

X-ray of SIJs revealed bilateral grade 3–4 sacroiliitis including subchondral sclerosis, obliteration of the SIJs and partial ankylosis, and ankylosis in the apophyseal joints of the lumbar vertebrae. Abdominal and thoracic computerized tomography (CT) revealed a thickened wall of terminal ileum and caecum and widespread lymph node enhancements of pathologic sizes in the intra-abdominal areas. An ileocolonoscopy showed linear ileocecal valve ulcers. Erythema and loss of vascular pattern of the mucosa because of intestinal wall oedema were shown in **Figure 1, 2 and 3**. Histopathological findings of the ileocecal valve showed intestinal peripheral T-cell lymphoma. TNFi was interrupted because of the possible progression of the lymphoma.

## LITERATURE SEARCH

A literature search was conducted in the PubMed database between January 1980 and November 2020. The keywords including ‘ankylosing spondylitis’ and ‘lymphoma’ were used. The available abstract and/or full text in English were examined. Twenty-five papers were retrieved with full text and/or abstract, among which all case reports were found with the search term “ankylosing spondylitis and lymphoma” in the same title, involving in total of 13 cases. Clinical characteristics (age and gender) provided various lymphoma types and ongoing treatment modalities of the previously published patients were detailed in **Table 1**.

**Table 1.** Clinical characteristics of the previously published patients.

**Author(s)**	**Year**	**Age, Gender**	**Patient (n)**	**NHL**	**HL**	**Drug use**
Jantunen E et al.^13^	2000	60, M	1	B-cell NHL	-	SLZ-MTX
Gau JP et al.^14^	2000	NA/NA	1	Angiotropic lymphoma	-	NA
Pavithran K^15^	2002	37, M	1	NHL	-	NA
Khan SY et al.^16^	2004	65, M	1	NHL	-	NA
Dauendorffer JN^17^	2007	NA/NA	1	Sezary syndrome	-	Infliximab
Aksu K et al.^18^	2007	NA/NA	1	-	HL	Etanercept
Sanli H et al.^19^	2007	32, M	1	Mycosis fungoides	-	Infliximab
Xu L et al.^20^	2011	45, F	1	Angioimmunoblastic T cell lymphoma	-	Etanercept
Aksu K et al.^11^	2011	NA/NA	1	NHL	-	Etanercept
Kim YS et al.^21^	2012	38, M	1	-	HL	None
Jung KH et al.^22^	2013	NA/NA	1	Angioimmunoblastic T cell lymphoma	-	Etanercept
Monti S et al.^23^	2016	52, M	1	B-cell NHL	-	Anti-TNF
Stoicanescu LD et al.^24^	2019	47, F	1	-	HL	SLZ-MTX-NSAID

NA: not available; F: female; M: male; NHL: non-Hodgkin’s lymphoma; HL: Hodgkin’s lymphoma; SLZ: salazopyrin; MTX: methotrexate; NSAID: nonsteroidal anti-inflammatory drug.

The most mentioned anti-TNF agents are etanercept and infliximab related to the development of lymphoma in AS patients in the literature. The vast majority of patients were also NHL.

## DISCUSSION

Although there is no clear increased cancer risk related to the disease course and immunosuppressive drugs in AS patients as mentioned above, some uncertainty is still present.^10^ This condition may be explained by chronic subclinical inflammation that occurs in the gut mucosa in 50–60% of AS patients in addition to TNFi use.^12^ There are accumulated case reports in the related literature over the recent years. After these subsequently published cases (**Table 1**), the authors can also keep in mind the possible development risk of lymphoma in these patients with considering the recent publication bias.

Rare cases like this patient lead us to emphasize some points. An interesting point of this case was that Crohn’s disease was initially suspected due to clinical symptoms, typical endoscopy findings, and many of related cases in the literature.^25^ The revelation of the underlying silent Crohn’s disease or togetherness of CD and AS was not considered due to highly effective treatment with infliximab, and no personal or family history of inflammatory bowel disease in the patient. However, anti-TNF drugs may induce the production of variable autoantibodies against themselves.^26^ Sazonov A et al. recently revealed a relationship between HLADQA1*05 carriages and the development of anti-drug antibodies to infliximab.^27^ Neutralizing IgG/IgE antibodies against infliximab may lower plasma drug concentrations and may cause the more active disease at the same dosages.^28^ Thus, clinicians have to prescribe higher drug doses to achieve remission or low disease activity. Such as in our patient, in the presence of red flags likewise weight loss, paradoxical adverse effects of anti-TNF agents should be finally considered after comprehensive diagnostic research.

Lesions of the terminal ileum and small intestine should not always be interpreted as Crohn’s disease. Investigation of the colon and/or rectum biopsies for occult Crohn’s disease may help clinicians differentiating from other diseases and prevent further research.^29^ It involves a variable differential diagnosis including tuberculosis and/or unusual infections, primary cancers and lymphomas of the intestine, metastases from other organs, Behçet’s disease and/or other vasculitides, and side effects of drugs.^30^ Neoplasms of the small intestine are uncommon and are often delayed due to nonspecific symptoms and findings, and is inaccessible by endoscopy.^31^ Variable radiographic patterns including circumferential lesion, cavitary lesion, and mesenteric nodal disease may be present for lymphoma.^32^ Those patients with colorectal and/or gastric involvement, diffuse macroscopic infiltration, perforation, and high-grade histology have poor prognoses whereas those with early-stage B-cell phenotype and radical tumour respectability are associated with a better survival rate.^33^ Luckily, the patient was presented with diarrhoea and ileocecal valve involvement. Examining pathologic biopsy specimens are gold standard criteria for diagnosis after clinicians take into consideration of lymphoma in the differential diagnosis and the presence of the symptoms compatible with the disease and non-response to therapeutic drugs.^34^

The therapeutic strategy in our patient included chemotherapy based on the disease extent, histologic type, and clinical stage with a favourable prognosis.
